# Parietal signatures of urge control in paediatric tic disorders: Hyperactivation and salience-biased coupling

**DOI:** 10.1007/s00787-026-03029-z

**Published:** 2026-04-10

**Authors:** Theresa V. Heinen, Julia Schmidgen, Stephan Bender, Kerstin Konrad

**Affiliations:** 1 Forschungszentrum Jülich GmbH (INM-10), Wilhelm-Johnen-Straße, 52428 Juelich, Germany; 2 Institute of Medical Psychology and Medical Sociology, Faculty of Medicine, RWTH Aachen University, 52074 Aachen, Germany; 3 Section Child Neuropsychology, Department of Child and Adolescent Psychiatry, Psychosomatics and Psychotherapy, University Hospital, RWTH Aachen, 52074 Aachen, Germany; 4 Department of Child and Adolescent Psychiatry, Psychosomatics and Psychotherapy, Faculty of Medicine and University Hospital Cologne, University of Cologne, 50931 Cologne, Germany

**Keywords:** Tic disorders, Premonitory urges, Urge suppression, Functional MRI, Functional connectivity

## Abstract

Premonitory urges (PU) are central to tic expression and guide behavioural treatment yet are difficult to measure in children using self-report because urge awareness often lags tic onset. We therefore examined neural correlates of urge control in paediatric tic disorders (TS) across natural urge conditions (blink, yawn) and tics, using an fMRI release-suppression task in 25 medication-naïve children and adolescents with TS (76% male; 7–16 years) and 25 matched typically developing control participants (76% male; 6–17 years). We compared natural urge suppression between the TS group and controls at behavioural and neural levels. In participants with TS, we identified neural correlates of voluntary tic suppression, and examined task-evoked connectivity during tic suppression using psychophysiological interaction (PPI) analyses with parietal seeds derived from natural urge effects to assess coupling with control/monitoring and interoceptive-salience networks. Across blink and yawn, TS participants showed greater suppression-related activation than controls in left posterior parietal association cortex, with additional right parietal and frontal engagement for yawn. Behavioural impairment was selective to blink. Tic suppression did not yield uniform group-level activation, but suppression success was positively associated with engagement of executive, salience, and default-mode hubs. PPI analyses showed that stronger parietal-cingulo-opercular coupling was negatively associated with suppression success, whereas fronto-parietal coupling showed no positive association. These network-level associations provide a framework for refining models of urge control in youth and motivate future studies with larger samples to evaluate their robustness and potential translational relevance.

**Supplementary Information:**

The online version contains supplementary material available at https://doi.org/10.1007/s00787-026-03029-z.

## Introduction

Tic disorders (TS), including Tourette syndrome, are neurodevelopmental conditions characterized by sudden, repetitive movements or vocalizations. These typically emerge between 6 and 8 years and peak around 10–12, highlighting childhood as a critical window for mechanistic insight [1, 2].

While tics have traditionally been classified as involuntary, empirical models increasingly conceptualize them as partially voluntary responses to involuntary internal sensations, referred to as premonitory urges (PU) [3–5]. PU are typically described as uncomfortable bodily experiences—such as tension, pressure, or itching—and are relieved transiently via tic expression, establishing a negative reinforcement loop [6–8]. Accordingly, behavioural interventions emphasize detection and regulation of PU [9]. Yet, PU phenomenology in youth remains poorly understood. Awareness typically emerges years after tic onset, and reported PU prevalence increases with age, though estimates vary considerably across samples and assessment methods [10]. In younger children particularly, categorical reports yield numbers substantially lower than scale-based assessments (46.7% in children < 10 years [11] vs. 81% in children ≤ 7 years [12]), suggesting developmental influences on subjective awareness and reporting of PU [10, 13]. Importantly, successful tic suppression has been observed even in children denying antecedent sensations, implying PU-related influences on behaviour outside of conscious awareness [11, 14]. Conversely, self-reported PU presence and intensity explain only a limited proportion of variance in tic severity and suppression ability in youth, with associations typically modest and varying across age groups and comorbidity profiles [10].

Taken together, this disconnect between subjective reports and behavioural control motivates investigation of mechanisms underlying urge-related behaviour. At the neural level, urge generation has been consistently linked to regions of the salience network, particularly anterior insula, and anterior cingulate cortex (ACC), which are implicated in the detection and prioritization of interoceptive and exteroceptive signals [15]. In TS, alterations in the intrinsic organization of this circuitry have been reported and proposed to reflect atypical sensory sensitivity or salience attribution, potentially increasing the influence of bodily signals on urge-related behaviour [16]. From this perspective, tic expression may arise from alterations in urge-related processing upstream of motor execution, rather than from motor output stages alone. Accordingly, if relief from PU reinforces tic learning, then targeting their neural generators – rather than downstream motor expressions – may yield more durable therapeutic benefit. This pattern is consistent with neuromodulation trials showing variable effects for stimulation of supplementary motor and premotor regions, whereas parietal stimulation has yielded more robust improvements in tic severity and PU [17].

Beyond local salience-network alterations, resting-state studies further report atypical connectivity both within fronto-parietal control systems and between salience and control networks [18, 19]. Within this altered architecture, successful tic regulation has been associated with compensatory recruitment of distributed monitoring resources, as paediatric data link successful suppression to enhanced fronto-parietal connectivity within default-mode-network (DMN) [20]. Adult tic suppression studies further implicate inferior frontal, premotor, temporal, and parietal regions, consistent with engagement of inhibitory control under concurrent urge drive [21, 22].

Despite providing important insights, tic suppression paradigms are inherently limited by their lack of comparability with typically developing individuals. Studying natural urges can offer complementary model systems. Owing to its repetitive, semi-voluntary nature, blink suppression is a well-established proxy for TS, revealing reduced suppression performance in TS alongside overactivations interpreted as compensatory recruitment of control resources [7, 22, 23]. Yawning, although less studied, may provide an additional proxy, sharing key features with tics: it is driven by gradually intensifying urges, subserved by overlapping interoceptive motor circuits, and can be reliably induced even in young children [24, 25]. Critically, few studies have compared natural urge suppression between participants with TS and typically developing controls [23, 26, 27], and none have examined multiple urge domains within the same cohort.

We employed a multi-domain fMRI design to (i) test whether natural urges are abnormally processed in paediatric participants with TS compared with matched typically developing controls at behavioural and neural levels; (ii) delineate neural substrates of pathological urges and their relation to voluntary tic control; and (iii) integrate these approaches using psychophysiological interactions to examine task-evoked connectivity during tic suppression, with seeds derived from alterations identified in (i). We hypothesized that participants with TS would show heightened interoceptive-salience responses accompanied by increased engagement of control/monitoring resources, together with lower suppression performance; that voluntary tic suppression would engage canonical inhibitory-interoceptive networks; and that stronger control/monitoring connectivity would relate positively, whereas interoceptive-salience connectivity would relate negatively to suppression success.

## Methods and materials

### Participants

The study involved 25 medication-naïve children and adolescents with Tourette syndrome or chronic tic disorder (age 7–16 years; M_age_ = 10.4, SD = 2.6, 76% male) and 25 typically developing controls (age 6–17 years; *M*_age_ = 10.6, SD = 3.0; 76% male; see Table 1), matched one-to-one to participants with TS based on age, sex, handedness, IQ, and depressive symptomatology. This control subsample was algorithmically drawn from a larger cohort of typically developing children and adolescents (*N* = 51; age 5–17 years; *M*_age_ = 10.9, SD = 3.2; 45% male), which also served as a robust normative reference for task activations. In the TS group, quality-control exclusions (device malfunctions, excessive motion) and procedural exclusions (premature termination) reduced availability of concurrently acquired behavioural and fMRI data (to varying degrees across task conditions): behavioural – blink *n* = 23, yawn *n* = 25, tic *n* = 19; fMRI – blink *n* = 16, yawn *n* = 13, tic *n* = 11 (see Supplementary Table S1). Matched control participants were excluded whenever the corresponding TS participant was excluded from a given analysis, ensuring that group comparability was preserved within each behavioural and fMRI analysis. Parents and adolescents (≥ 12 years) completed a structured interview (K-DIPS) to confirm diagnostic criteria in the TS group and absence of neuropsychiatric conditions in controls [28]. Exclusion criteria included: (i) full-scale IQ < 70, (ii) history of neurological disorders (i.e., epilepsy), (iii) premature birth (≤ 31 weeks), (iv) uncorrectable vision impairments, (v) MRI contraindications, or (vi) current/past use of psychoactive medication. The study was approved by the University Hospital Cologne ethics committee (medical faculty) and conducted in accordance with the Declaration of Helsinki. Informed consent and assent were obtained from parents and participants, respectively. All participants received financial compensation.

**Table 1:** Demographic and psychometric characteristics in the TS group and matched typically developing controls

Sample Characteristics	Controls (*N* = 25)	TS (*N* = 25)	Statistic	*P*
Age (M ± SD, range)	10.6 (± 3.0; 6–17)	10.4 (± 2.6; 7–16)	t (48) = -0.222	0.826
Male sex [n (%)]	19 (76)	19 (76)	x^2^ (1) = 0.000	1.000
Right handedness [n (%)]	23 (92)	25 (100)	x^2^ (1) = 2.083	0.149
IQ (M ± SD)	112.0 (± 12.3)	108.6 (± 12.5)	t (46) = -0.924	0.360
DIKJ total symptom score (M ± SD)	8.2 (± 4.7)	8.9 (± 5.7)	t (48) = 0.488	0.627
YGTSS total tic score (M ± SD)		17.8 (± 6.7; 7–34)		
PUTS urge intensity score (M ± SD; range)		19.1 (± 6.0; 9–30)		
Age at tic onset (M ± SD)		5.3 (± 1.6)		
Duration of tics [years (M ± SD; range)]		4.8 (± 2.8; 1–10)		
Tourette Syndrome [n (%)]		23 (92)		
Comorbid ADHD [n (%)]		6 (24)		

*Note*. Handedness assessed with the Edinburgh Handedness Scale [29]. General IQ based on Wechsler Intelligence Scale for Children – 5th Edition (WISC-V) [30]. *DIKJ* revised German version of Children’s Depression Inventory (CDI) [31]. *YGTSS* Yale Global Tic Severity Scale [32]. *PUTS* Premonitory urges for tics scale [12]. *M* Mean, *SD* Standard deviation.

### fMRI paradigm

**Fig. 1:**
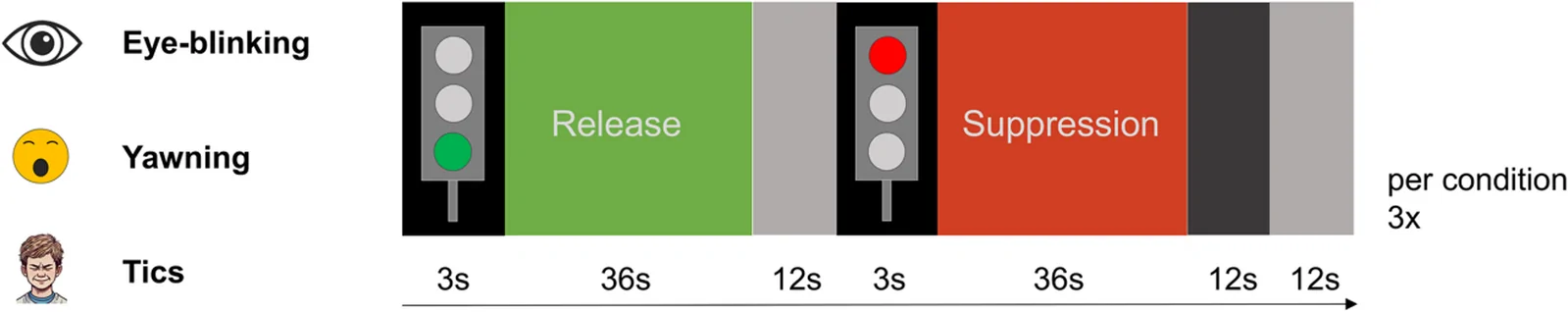
fMRI task paradigm Note Block design for voluntary release and suppression of blinks, yawns, and tics. Green/red traffic-light cues indicated task demand. Each condition comprised repeated release-suppression cycles separated by fixation (light grey); participants rated suppression difficulty after each suppression block (dark grey)

To examine voluntary urge suppression, we employed a block-design release/suppression paradigm across three domains: eye-blinking, yawning, and tics (TS group only) – including 6 × 36-s blocks per domain (3 release/3 suppression; green/red traffic-light cues; see Fig. 1). During release blocks, participants were instructed to allow the urge-related behaviour to occur spontaneously. During suppression blocks, they were instructed to inhibit the behaviour for the entire block duration and immediately resume suppression in case of escape events. Each suppression block was followed by a 5-point difficulty rating, with 12-s fixation baselines between blocks. Domains were presented in a fixed order (blink, yawn, tic). Visual stimulation consisted of actor videos during blink (eye region only) and yawn (head/upper torso) blocks, and self-videos (head/upper torso) recorded immediately before scanning during tic blocks to potentiate urges [33].

### Statistical analysis of behavioural data

Blinks, yawns, and tics were recorded using an MR-compatible eye-tracker/video camera and documented offline by two independent raters using the Behavioural Observation Research Interactive Software [34], yielding excellent interrater reliability (ICC: blinks = 0.990, yawns = 0.947, tics = 0.953; *p* < .001). For yawning, given low base rates of events, success was indexed as the raw difference in event count (release − suppression). For blinking and tics, success was quantified as the percentage reduction in event frequency. Subjective difficulty was averaged across suppression blocks. Within-group suppression effects (suppression < release) were assessed using Wilcoxon signed-rank tests; between-group differences in suppression success and perceived difficulty using non-parametric Mann-Whitney U tests. To examine clinical relevance, we ran Kendall’s Tau-b correlations between suppression success, tic severity (YGTSS Total Tic Score) and PU intensity (PUTS), and additionally tested the association between PU intensity and perceived suppression difficulty. We also explored cross-domain associations in suppression success. All analyses were run in SPSS (30.0.0) with α = 0.05 (two-tailed).

### fMRI acquisition and preprocessing

To minimize in-scanner motion, participants completed mock-scanner training with real-time motion feedback and were stabilized with foam padding within the head coil. MRI data were acquired at Research Centre Juelich using a 3T Siemens MAGNETOM Prisma scanner (Siemens Healthcare, Erlangen, Germany) with a 64-channel head coil. T1-weighted anatomical images were collected using a magnetization-prepared rapid gradient echo (MP-RAGE) sequence (TR = 1790 ms, TE = 2.53 ms, flip angle = 8°, 176 slices, slice thickness = 0.9 mm, interslice gap = 0.45 mm, FOV = 256 mm, voxel size = 0.9 × 0.9 × 0.9 mm). Functional images were acquired using a T2*-weighted echoplanar imaging (EPI) sequence with multiband acceleration and blood oxygen level-dependent (BOLD) contrast (TR = 980 ms, TE = 30 ms, flip angle = 70°, 64 interleaved axial slices, slice thickness = 2.0 mm, interslice gap = 0.2 mm, FOV = 207 mm, voxel size = 2.2 × 2.2 × 2.0 mm). Preprocessing and fist-level analyses were performed using SPM12 (Wellcome Centre for Human Neuroimaging, London, UK), implemented in MATLAB (The MathWorks, Natick, MA, USA). Functional time series were realigned and unwarped to correct for motion. Runs with translational displacement exceeding 2.2 mm (one voxel) or framewise shifts greater than 1.1 mm (half a voxel) were excluded, consistent with developmental fMRI motion criteria [35]. Images were then slice-time corrected, co-registered to the T1, normalized to MNI space (unified segmentation), and smoothed with an 8-mm FWHM Gaussian kernel.

### fMRI statistical analysis

####  First-level modelling

 Voxelwise general linear models (GLMs) were fit per urge domain, with regressors (instruction, baseline, release, suppression, rating) convolved with the canonical hemodynamic response function (HRF). To improve specificity of suppression-related activity, escape events were excluded from suppression regressors [22]. For blinks, we modelled either escape-free suppression blocks or uninterrupted segments ≥ 2.5× the subject’s mean inter-blink-interval (total release-block duration ÷ total release-block blinks). Because blinks are rapid and low-motion, we additionally estimated a blink model retaining escapes to preserve unfragmented suppression periods (task-recruitment check only) [36]. For yawns, full onset-offset of escapes was removed from suppression regressors. As tics are highly heterogeneous in temporal structure/often occur in bursts (precluding reliable inter-tic-intervals), tic epochs were excluded from suppression regressors based on frame-by-frame video annotation [7].

####  Group-level inference

 First-level contrast images (suppression > release) were entered into nonparametric permutation inference (10,000 permutations; cluster-forming threshold *p* = .001; variance smoothing 8 mm; cluster-level FWE α = 0.05) using the SnPM13 Toolbox, yielding statistical maps expressed as pseudo-t values (www.nisox.org/Software/SnPM13/) [37]. This approach was chosen to accommodate modest sample sizes, unequal variances between groups (arising from differences in suppression success), and potential non-normal residuals. Clusters were labelled using the SPM Anatomy Toolbox (cytoarchitectonic maximum-probability maps) [38]. To establish task recruitment, we ran one-sample t-tests per urge type in the TS group and the large typically developing cohort. Between-groups differences (blink, yawn) were tested with two-sample t-tests (TS group vs. matched controls drawn from the larger pool). For tic suppression, we additionally performed simple regression relating suppression-related activation to suppression success.

### Psychophysiological interaction (PPI) analysis

To estimate functional coupling during voluntary tic suppression, we used PPI (context-dependent seed-based connectivity) [39, 40] with two parietal seeds derived from group-level TS > control contrasts during natural urge suppression (blink: left superior parietal lobule; yawn: right inferior parietal/angular gyrus). For each seed, an 8-mm sphere was centred on the group peak. Within this sphere, each participant’s nearest suprathreshold maximum was identified (first-level GLM; initial *p* < .05; relaxed in 0.05 steps if necessary) and the first eigenvariate extracted (adjusted for effects of interest). The physiological seed time series was multiplied with the psychological regressor (suppression > release) to generate the PPI interaction term. First- level GLMs were re-estimated including this interaction term alongside original task regressors and motion parameters as covariates of no interest. PPI contrast images were entered into group-level inference in SnPM13 (10,000 permutations; cluster-forming threshold *p* = .001; variance smoothing 8 mm; cluster-level FWE α = 0.05). Per seed, we assessed group-mean context-dependent coupling (one-sample t-tests) and associations with tic suppression success (simple regressions). Hypothesis-constrained tests used two a priori masks: (i) a control/monitoring (DMN-linked) mask including medial prefrontal/superior frontal gyri (BA10, BA32) and posterior cingulate cortex for positive (control-enhancing) associations; and (ii) an interoceptive-salience (cingulo-opercular) mask including insula (Areas Id1–7), parietal operculum (OP1–4), SMA (Area 6mc), and dACC/pre-SMA (Areas p24ab, 24c, p32, 6mr) for negative (performance-impairing) associations. Exploratory whole-brain PPIs were performed additionally.

## Results

### Natural urge suppression

#### Task recruitment

We only briefly report task-activations for natural urge suppression (large typically developing cohort; TS sample), to provide a robust normative reference and document that the paradigm elicited expected patterns. During blink suppression (escapes retained), both the typically developing cohort (*n* = 51) and the TS group (*n* = 16) engaged an inhibitory-interoceptive control ensemble bilaterally, including inferior frontal gyrus (IFG), frontal operculum (FOp) and dorsal anterior insula (dAI), with additional DLPFC engagement. In contrast, yawn suppression recruited broader network activations, encompassing frontal, parietal, temporal, and midline regions in both the typically developing cohort (*n* = 39) and the TS group (*n* = 13). For contrast maps and cluster details see supplementary material (Figure S1). In the following, we report TS–control contrasts using matched subsamples drawn from the typically developing cohort.

#### Behavioural performance

 Participants with TS and matched typically developing controls successfully reduced blinking and yawning during suppression relative to release blocks (Blink: TS: Z = -4.20, *p* < .001, *r* = − .62; controls: Z = -4.20, *p* < .001, *r* = − .62; Yawn: TS: Z = -3.42, *p* < .001; controls: Z = -3.54, *p* < .001). Between groups, the TS group showed significantly reduced blink suppression success compared to controls. Yawn suppression showed no group difference (see Table 2). Subjective difficulty was comparable between groups and unrelated to task performance (Blink: TS: Mdn = 2.33, t_b_ = − 0.004, *p* = .977; controls: Mdn = 1.67, t_b_ = − 0.045, *p* = .781; U = 324.000, Z = 1.628, *p* = .103; Yawn: TS: Mdn = 1.00, t_b_ = 0.036, *p* = .839; controls: Mdn = 1.00, controls: t_b_ = 0.140, *p* = .411; U = 259.500, Z = − 0.677, *p* = .499).

**Table 2:** Task performance parameters in the TS group and matched typically developing controls

Condition	Group	Events Release (Mdn; IQR)	Events Suppression (Mdn; IQR)	Suppression success (Mdn; IQR)	Between-Group Comparison (Suppression success)
Blinking	TS (*N* = 23)	19.33; 14.17	5.67; 5.50	69.00; 21.00	U = 125.000; Z = -3.070; *p* = .002
	C (*N* = 23)	11.67; 10.33	1.00; 4.00	85.00; 25.00	
Yawning	TS (*N* = 25)	0.33; 1.33	0.00; 0.00	0.33; 1.25	U = 303.500; Z = -0.180; *p* = .857
	C (*N* = 25)	0.67; 1.00	0.00; 0.00	0.67; 1.00	
Tic	TS (*N* = 19)	5.67; 3.67	2.00; 2.33	61.00; 21.00	-

*Note*. For yawning, suppression success = raw difference in event count (release – suppression); for blinking and tics, suppression success = percentage reduction in event frequency. Values were considered statistically significant at *p* < .05. *TS* participants with chronic tic disorders, *C* typically developing controls. *Mdn* Median, *IQR* Interquartile range. Behavioural blink data were previously reported in a smaller, partially overlapping sample [41].

#### Between-groups fMRI contrasts

**Fig. 2:**
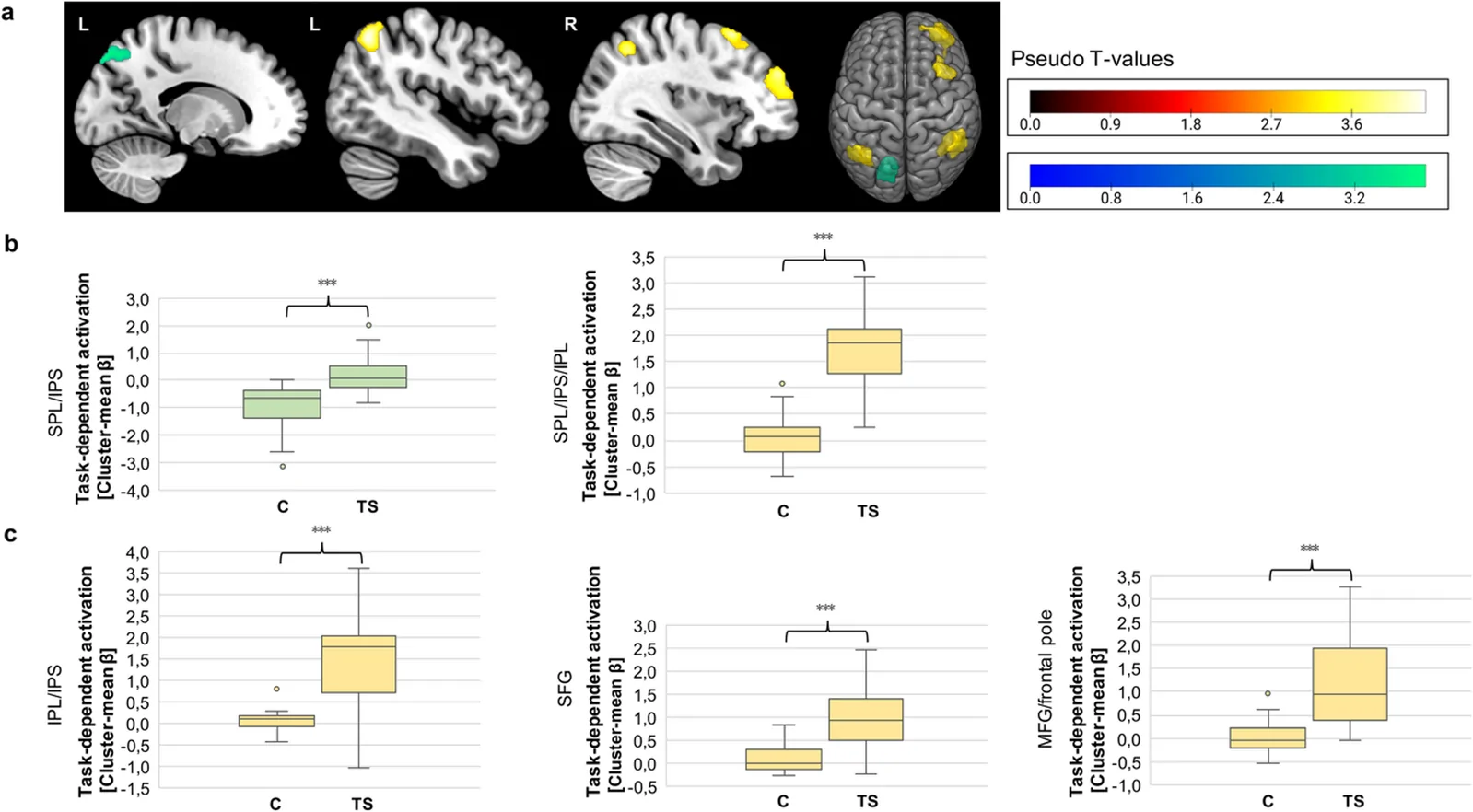
Neural overactivation in paediatric TS during natural-urge suppression Note **(a)** Between-group contrast maps show greater activation in participants with TS compared to matched typically developing controls (C) during blink (green) and yawn (yellow) suppression. Colour bars indicate pseudo-t values. Boxplots correspond to clusters shown in (a); **(b)** = left hemisphere, **(c)** = right hemisphere. Boxes indicate median and interquartile range (IQR); whiskers denote 1.5 × IQR; circles = outliers; asterisks = *p* < .001. L = left; R = right.

 During blink suppression (escapes removed), participants with TS (*n* = 16) showed significantly greater activation than matched typically developing controls (*n* = 16) in a left-hemispheric posterior-parietal cluster encompassing superior parietal lobule (SPL) and intraparietal sulcus (IPS), extending into the precuneus and lateral occipital cortex (x, y, z = -20, -74, 50; k_E =_ 442; pseudo t = 3.92; p_FWE−corr_. = 0.028). No significant group differences were observed in the opposite contrast (controls > TS). During yawn suppression, TS participants (*n* = 13) showed significantly greater activation than matched controls (*n* = 13) in four clusters. These included a left-hemispheric parietal cluster centred at the boundary between superior and inferior parietal lobules (x, y, z = − 36, − 62, 60; k_E_ = 484; pseudo-t = 4.45; p_FWE−corr_. = 0.024), a right-hemispheric parietal cluster located in inferior parietal (IPL) and angular regions, extending into IPS (x, y, z = 42, − 52, 52; k_E_ = 747; pseudo-t = 4.24; p_FWE−corr_. = 0.014), a smaller cluster in the right superior frontal gyrus (SFG; x, y, z = 36, 18, 58; k_E_ = 248; pseudo-t = 4.40; p_FWE−corr_. = 0.047), and a cluster in the right frontal pole/middle frontal gyrus (MFG; x, y, z = 34, 52, 30; k_E_ = 601; pseudo-t = 4.07; p_FWE−corr_. = 0.019). No significant effects were observed in the opposite contrast. For contrast maps see Fig. 2.

### Pathological urge suppression

#### Behavioural performance 

Tic frequency was significantly reduced during suppression relative to release blocks (Wilcoxon signed-rank test: Z = -3.824, *p* < .001; see Table 2). Suppression success was unrelated to tic severity (t_b_ = − 0.163, *p* = .342) or premonitory urge intensity (t_b_ = 0.193, *p* = .260). Perceived suppression difficulty likewise did not correlate with urge intensity (t_b_ = 0.166, *p* = .332). No significant correlations were found between suppression success across natural and pathological urge types (tic vs. blink: t_b_ = − 0.173, *p* = .308; tic vs. yawn: t_b_ = − 0.135, *p* = .585).

#### Neural substrates of tic suppression

**Fig. 3:**
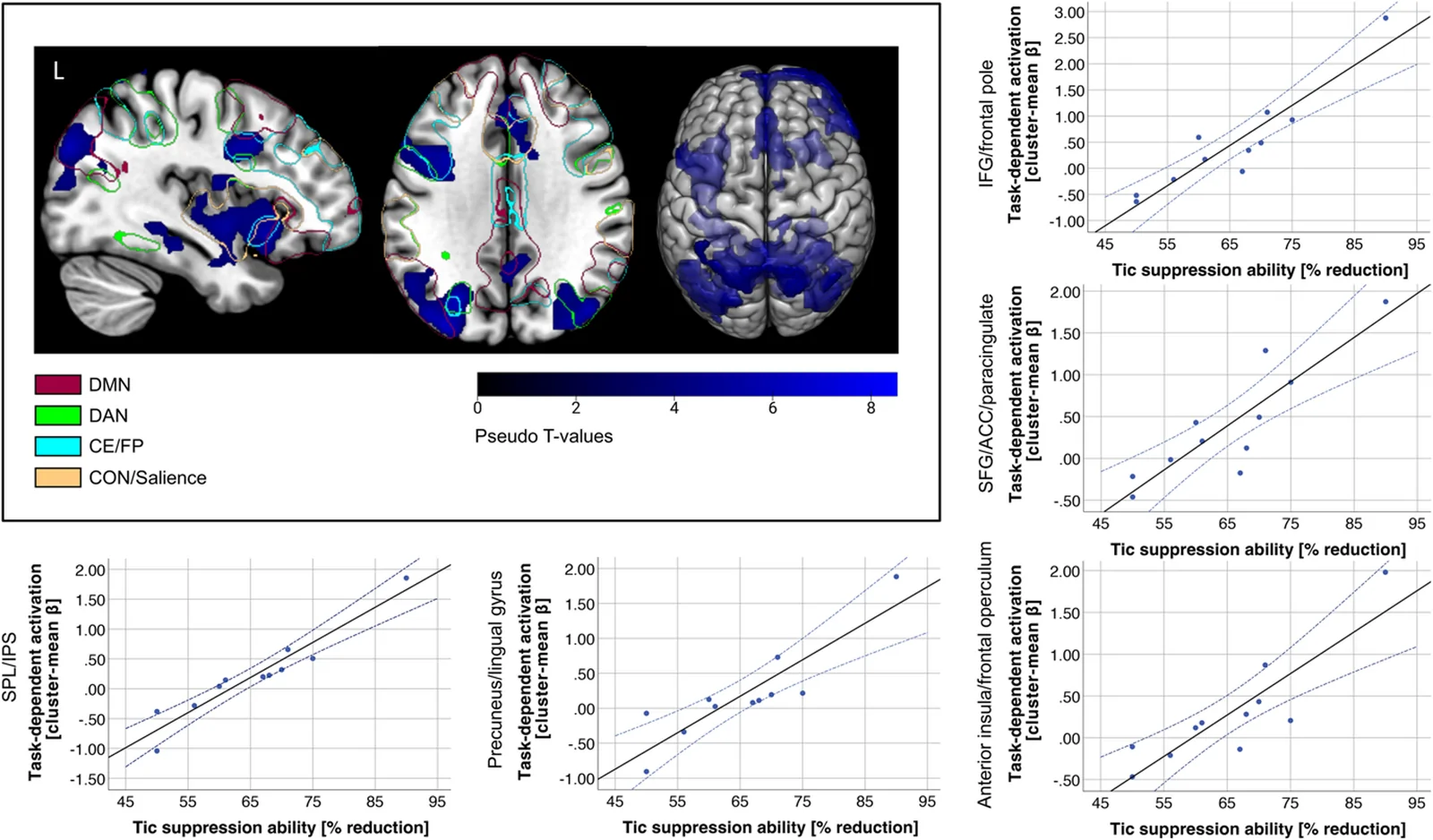
Neural correlates of tic suppression success Note Whole-brain regression maps depict positive associations between tic suppression success (% reduction) and activation during tic suppression (suppression > release). Colour bars indicate pseudo-t values. Network outlines: Schaefer2018 400 × 7 parcellation (mapped to Yeo-7); default-mode network (DMN, maroon), dorsal-attention network (DAN, green), central-executive/fronto-parietal network (CE/FP, cyan), cingulo-opercular network (CON/Salience, orange). Scatterplots correspond to significant clusters shown in the regression maps; each data point represents one participant. Solid lines = least-squares fit; dashed lines = 95% confidence intervals. L = left.

 In participants with TS (*n* = 11), no significant group-mean activation was observed for tic suppression (escapes removed) relative to release. However, activation showed positive associations with suppression success across a distributed set of regions (see Fig. 3). The largest cluster spanned bilateral posterior parietal-occipital cortex, including SPL, IPS, inferior parietal lobule (IPL), and superior lateral occipital cortex. In the left hemisphere, it extended anteromedially into posterior cingulate (PCC), hippocampus and putamen (x, y, z = 18, − 70, 60; k_E_ = 10755; pseudo-t = 8.63; p_FWE−corr_. = 0.003). Additional posterior success-linked effects emerged in lingual gyrus/precuneus with hippocampal involvement (x, y, z = 12, − 52, 0; k_E_ = 2134; pseudo-t = 6.00; p_FWE−corr_. = 0.011), and in inferior lateral occipital cortex (x, y, z = 50, − 70, − 6; k_E_ = 426; pseudo-t = 4.72; p_FWE−corr_. = 0.043). Beyond this posterior complex, positive associations were observed in frontal executive and salience regions, including frontal pole, IFG/dACC (x, y, z = 38, 64, 8; k_E_ = 1522; pseudo-t = 6.22; p_FWE−corr_. = 0.015), SFG/paracingulate cortex (x, y, z = 0, 36, 40; k_E_ = 832; pseudo-t = 5.69; p_FWE−corr_. = 0.026), and right dAI/FOp (x, y, z = 32, 18, − 6; k_E_ = 521; pseudo-t = 5.59; p_FWE−corr_. = 0.038).

### Psychophysiological interactions

#### Group-level effects

 No significant task-related coupling changes (suppression > release) were observed for either seed (mask-constrained or whole-brain).

#### Associations with suppression success

**Fig. 4:**
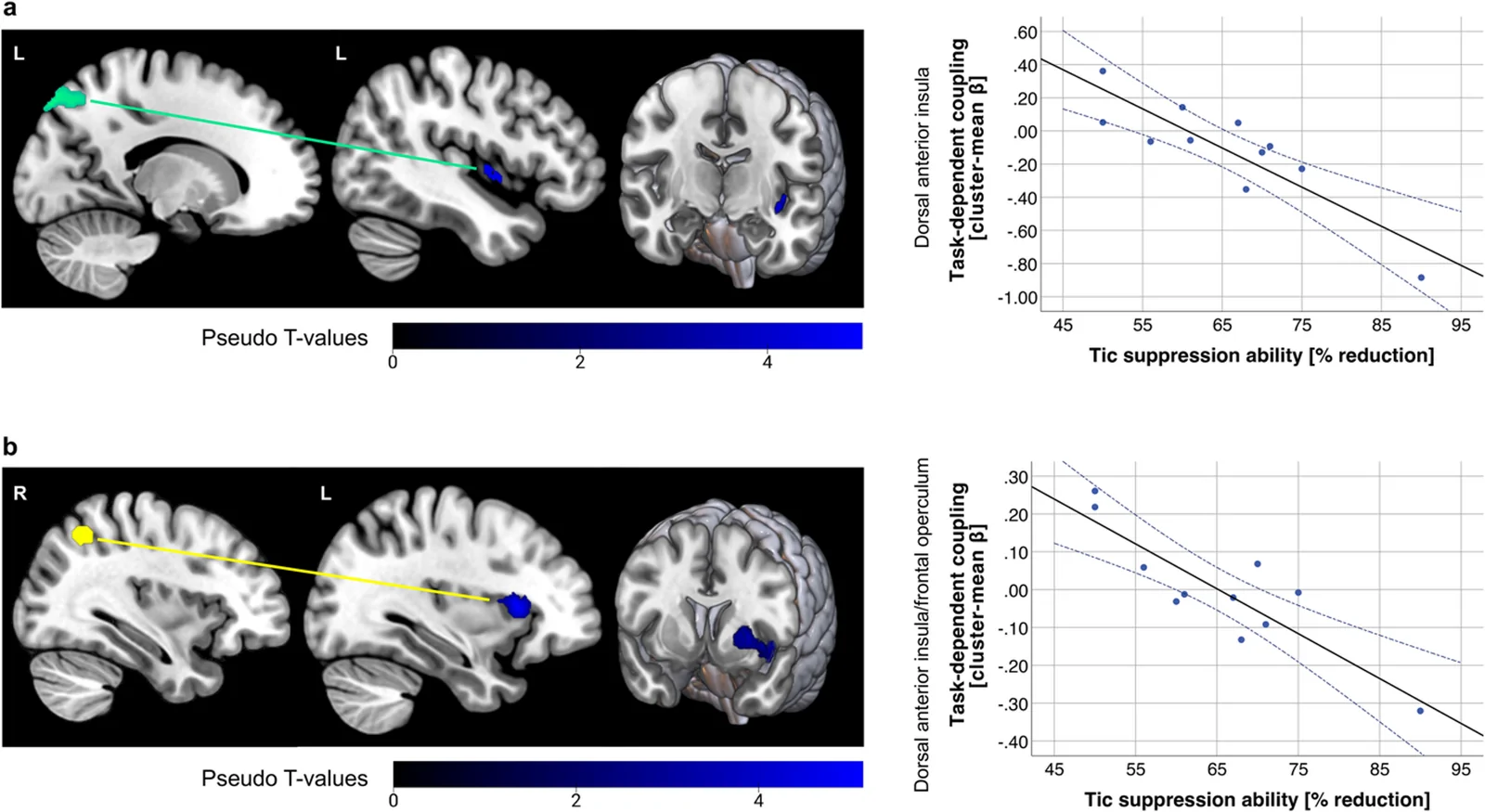
Negative association between parietal–salience coupling and tic suppression success Note **(a)** Left-hemispheric parietal seed (green) derived from the blink TS > control contrast. **(b)** Right-hemispheric parietal seed (yellow) derived from the yawn TS > control contrast. PPI regression maps show regions within the interoceptive-salience mask whose coupling with the respective parietal seed was negatively associated with tic suppression success. Scatterplots: each data point represents one participant. Solid lines = least-squares fit; dashed lines = 95% confidence intervals. Colour bar indicates pseudo-t values. L = left; R = right.

 No positive associations emerged between suppression success and coupling within the control/monitoring mask for either seed. In contrast, negative associations (i.e., stronger coupling associated with poorer suppression) emerged within the interoceptive-salience mask for both seeds (see Fig. 4). For the right-hemispheric IPL/angular seed (from yawn), suppression success was negatively related to coupling with dAI and FOp (x, y, z = − 32, 18, 8; k_E_ = 235; t = 5.25; p_FWE−corr_. = 0.018). For the left-hemispheric SPL seed (from blink), a small focal effect was observed in dAI (x, y, z = − 44, − 2, − 2; k_E_ = 23; t = 5.24; p_FWE−corr_. = 0.017); evident at voxel-level inference but not exceeding permutation-based cluster-extent thresholds. No positive whole-brain associations were detected for either seed. For the right-hemispheric IPL/angular seed, negative associations were observed with left cerebellar crus (x, y, z = − 34, − 70, − 36; k_E_ = 4428; t = 7.42; p_FWE−corr_. = 0.011) and extrastriate-visual cortex (x, y, z = − 8, − 70, 20; k_E_ = 3323; t = 7.15; p_FWE−corr_. = 0.014) (see Figure S2). No additional whole-brain effects were detected for the left-hemispheric SPL seed.

## Discussion

This study showed that participants with TS exhibited parietal hyperactivation across natural urge suppression, with behavioural impairment for blink and additional frontal hyperactivation for yawn. Tic suppression lacked uniform group effects, but suppression success scaled positively with engagement of executive, salience, and DMN-linked hubs; and negatively with parietal–salience coupling. Importantly, because tic suppression fMRI and connectivity analyses were based on a relatively small sample, these regression- and PPI-based associations should be interpreted as preliminary. Within this constraint, the present findings identify parietal cortex as a recurring and prominently engaged node across urge-related contexts in paediatric TS and suggest that variability in tic suppression success may relate not only to regional activation but also the configuration of parietal interactions with salience nodes.

### Fronto-parietal overactivations during natural urge suppression in TS

Across natural urge domains, participants with TS exhibited greater suppression-related activation than matched typically developing controls in frontal and parietal association cortices. During blink suppression, overactivation emerged in a left-lateralized cluster encompassing SPL, IPS, precuneus, and lateral occipital cortex. A highly similar posterior-parietal cluster involving SPL/IPS was overrecruited during yawn suppression, indicating that between-group differences are shared across distinct natural urge modalities. Additional yawn suppression overactivations emerged in right-hemispheric parietal (IPL/IPS/angular gyrus) and frontal regions (frontal pole/SFG/MFG). All these between-group differences were specific to instructed suppression periods, with no corresponding effects during release blocks, linking them to task-related suppression demands rather than baseline group differences (e.g., tic-related activity) [42].

The parietal-occipital blink suppression pattern observed here, together with behavioural deficits in the TS group, closely resembles findings from blink suppression in participants with obsessive-compulsive disorder, another condition characterized by urge-driven behaviours, where parietal and occipital hyperactivation was interpreted as reflecting heightened visuospatial attentional effort in the context of poorer suppression performance [43]. Consistent with this demand account, a study in typically developing participants previously reported greater suppression-related parietal activity for the subgroup of poorer blink suppressors [44]. Although prior blink suppression studies in participants with TS reported activation increases in partially different regions (e.g., frontal/cingulate, or temporal cortex) [23, 26], these were similarly interpreted in terms of difficulties with urge suppression and reallocation of attentional resources, with compensatory recruitment as a possible contributing factor. However, the present data do not permit determining whether such overactivations primarily index increased task demands or attempted compensation for impaired control. Together, these findings converge on the observation that blink suppression in clinical groups prone to urge-related behaviours is characterized by behavioural deficits alongside increased recruitment of task-relevant cortical resources.

Importantly, despite shared parietal overactivation across urge types, behavioural impairment emerged only for blink suppression, likely reflecting its particular difficulty in TS given shared effector pathways between ocular tics and voluntary blinks [7]. The presence of comparable parietal hyperactivation during yawn suppression, where performance was preserved, suggests that these effects cannot be explained solely by overt task difficulty or behavioural failure. At the same time, the present design does not permit disentangling suppression effort from processes related to urge build-up, as both are likely to co-vary over sustained suppression [27]. However, contrary to our a priori hypothesis of heightened interoceptive-salience engagement in participants with TS, we did not observe greater activation in core salience hubs typically implicated in urge generation (e.g., anterior insula, ACC) compared with controls [25]. Taken together, across natural urge modalities, group differences in the present paradigm were expressed most consistently within posterior parietal association regions, pointing to altered engagement of parietal systems during the suppression of urge-driven action tendencies in participants with TS. Notably, parietal differences have also been reported in TS across task contexts in which externally driven signals are behaviourally linked to action tendencies [45–47]. While these paradigms differ substantially from urge suppression, this convergence does not necessarily imply a unitary or disorder-specific mechanism but instead situates the present findings within a broader pattern of parietal involvement across diverse action-relevant contexts.

### Tic suppression in youth: variable access to multi-network resources

Unlike natural urge suppression, eliciting robust group-level activations, tic suppression did not yield uniform group-level effects. Activation instead scaled positively with individual tic suppression success: better suppressors showed stronger recruitment of frontal executive (frontal pole, DLPFC, IFG), parietal association (SPL/IPS/IPL), midline salience-related (dACC, dAI, FOp), DMN-linked (precuneus, hippocampus), and occipital cortices. This heterogeneity aligns with developmental accounts of tic awareness and volitional control, indicating that at this developmental stage, not all children with TS may have consolidated the neural and strategic mechanisms required for reliable tic suppression [6, 13, 48]. Additionally, interindividual variation in tic topography, frequency, and severity likely makes tic suppression a less clearly delineated control goal than natural urge suppression, further contributing to heterogeneous network recruitment across the TS group [49]. Consistent with this heterogeneity, suppression success did not correlate across urge types, suggesting that they rely on distinct control strategies that are variably effective within individuals. This dissociation is conceptually robust for blink and tic suppression, which were quantified using the same behavioural metric but interpretation of cross-domain numerical correlations involving yawns is limited by low event rates and the use of raw differences scores, which reduce interindividual variability relative to percentage-based measures. It should also be considered that tic suppression always occurred at the end of the scanning session and may therefore have been more susceptible to late-session factors such as fatigue or learning, which could have contributed to increased interindividual variability in tic suppression responses, potentially affecting both group-level tic suppression effects and between-domain differences. However, analyses of subjective difficulty ratings in participants with TS (see Supplement) indicated that perceived difficulty varied across domains in a quadratic pattern, with blink rated as most difficult, yawn as least difficult, and tic intermediate, inconsistent with a progressive increase in difficulty expected under accumulating fatigue or with a progressive decrease suggestive of simple habituation or learning effects.

Interestingly, the network profile observed in better suppressors overlaps regions implicated in adult tic suppression [22, 50], suggesting that as control strategies consolidate, recruitment patterns may increasingly converge toward adult-like configuration. Finally, although PUTS scores showed no association with activation, suppression success, perceived difficulty, or tic severity, suppression success nonetheless scaled with engagement of cingulo-opercular/salience regions, indicating that salience-related signals may be behaviourally relevant for tic control even when not captured by self-report measures. Taken together, these preliminary findings suggest that successful tic control in paediatric TS may rely on coordinated engagement of cingulo-opercular systems detecting urge salience and signalling control demands [13, 42, 51], DMN-linked nodes supporting self-monitoring/strategy maintenance [20, 52], and fronto-parietal/dorsal attention systems implementing adaptive top-down regulation and sustained attention [53]. Notably, several of the networks implicated here have also been reported to show alterations in paediatric ADHD, both at rest and during cognitive control tasks [54, 55], raising the possibility that comorbid ADHD could modulate tic suppression-related network recruitment and suppression success. However, despite subjective reports of poorer suppressibility, neither behavioural nor neuroimaging studies have found objective [20] tic suppression ability to be associated with ADHD comorbidity or symptom severity [11, 56, 57]. Accordingly, we observed no obvious clustering of participants with ADHD at extremes of suppression success or neural effects, although the small number of TS+ADHD participants precluded formal subgroup analyses.

### Integrating activations and connectivity: network segregation as a control principle?

Across natural and pathological urges, parietal cortex emerged as a central but ambivalent hub. It was robustly engaged and overactivated in the TS group relative to controls during natural urge processing, and positively scaled with tic suppression success. Connectivity analyses were therefore used to investigate aspects of parietal involvement that cannot be inferred from regional activation alone. Within this framework, stronger parietal coupling with cingulo-opercular/salience regions (insula/FOp) was shown to be inversely related to tic suppression success. This pattern was evident for both parietal hubs examined, though emerging more robustly for the right-hemispheric DMN-linked hub. Exploratory analyses further linked stronger parietal connectivity with cerebellar and extrastriate visual regions, previously implicated in tic generation [42], to poorer suppression. These preliminary findings extend resting-state reports, by suggesting that altered cingulo-opercular coupling in TS may also manifest in a task-evoked and performance-associated manner during active suppression [16, 18]. By contrast, we did not replicate prior accounts of adaptive fronto-parietal coupling [19, 20, 49], potentially reflecting developmental heterogeneity. In younger participants with TS, DMN contributions to tic control may be variable or strategy-dependent, yielding weak group-level PPI effects. Alternatively, DMN support could be more tonic than phasic, and thus less detectable with PPI. Notably, activation analyses still showed engagement of anterior and posterior DMN hubs, consistent with compensatory scaffolding for immature fronto-parietal systems. Throughout this discussion, fMRI tic suppression-related effects are framed in terms of sustained task demands to inhibit tics. However, interpretation of activation-success and PPI-success associations requires consideration of what suppression-related estimates reflect in the present design. Because tic epochs were excluded from suppression regressors, these estimates primarily reflect tic-free intervals, which do not uniquely reflect suppression effort, as they may also arise when tic-generating drive is momentarily low or insufficiently provoked. Accordingly, success associations are interpreted here as reflecting neural configurations associated with maintaining tic-free outcome states. Even when tic absence is partly driven by reduced urge-related drive (whether intrinsic or regulation-mediated), these states still index desirable network configurations during active urge provocation. Overall, the present findings motivate a network segregation perspective, in which effective urge control may depend on limiting parietal coupling with interoceptive-salience and associative/motor streams while allowing flexible recruitment into executive/monitoring configurations.

### Clinical implications and limitations

Interestingly, low-frequency rTMS targeting bilateral parietal cortex was previously reported to reduce both tic severity and PU [58], highlighting parietal hubs as promising targets. Extending this, a DBS network-mapping approach identified nodes along the cingulo-opercular/somato-cognitive action network axis (CON-SCAN) as potential multifocal targets suitable for non-invasive stimulation (e.g., rTMS, tDCS) [59]. These loci overlap with regions that emerged as relevant to tic suppression here, and more broadly, the present findings motivate further work to investigate connectivity-informed targets for multifocal neuromodulatory approaches that can act on distributed network dynamics rather than isolated sites. Importantly, our findings should be interpreted with caution owing to modest sample sizes, particularly for tic suppression. Mean tic severity in the present cohort was mild-to-moderate, raising the possibility that such milder symptom expression partly reflects relatively preserved tic suppression capacity. However, suppression success varied widely across participants and was unrelated to tic severity in our sample, arguing against uniformly enhanced control. Consistent with this, existing behavioural work in youth reports only modest and inconsistent associations between tic severity, premonitory urges, and suppression ability, indicating that symptom burden alone does not straightforwardly determine control capacity [10]. It nonetheless remains possible that more severe presentations would exhibit more pronounced neural alterations, for example reflecting stronger bottom-up salience/interception drive, or less efficient recruitment of executive networks. Task domains were presented in a fixed order, which can introduce potential confounds related to fatigue, habituation, or motion drift, although analyses of subjective difficulty ratings did not indicate progressive increases or decreases over time. Finally, PPI indexes task-dependent covariance rather than directionality, precluding causal claims about information flow. Larger paediatric cohorts with sufficient sampling across severity levels and developmental stages are required to establish the robustness and translational relevance of these network-level findings.

## Supplementary Information

Below is the link to the electronic supplementary material.

Supplementary Material 1

## Data Availability

The data underlying this study are available in the CRC1451 data registry (https://www.crc1451.uni-koeln.de/) and can be accessed upon reasonable request by contacting the corresponding author.
