# Supplementary material for: Parietal signatures of urge control in paediatric tic disorders: Hyperactivation and salience-biased coupling
Source: Eur Child Adolesc Psychiatry. 2026 Apr 10;35(8):2511–23. doi: 10.1007/s00787-026-03029-z (PMC13652861; doi:10.1007/s00787-026-03029-z)
Supplement: Supplementary file 1 [file 787_2026_3029_MOESM1_ESM.docx]

**Supplementary Material**

**Table S1** Final analyzed sample sizes and exclusions per task and modality (TS group)

| **Domain** | **N scanned** | **Behavioural exclusions (reason)** | **Behavioural**  **final N** | **Additional fMRI exclusions (reason)** | **fMRI**  **final N** |
| --- | --- | --- | --- | --- | --- |
| **Blink** | 25 | 2 (eye-tracker malfunction) | 23 | 7 (excessive motion) | 16 |
| **Yawn** | 25 | 0 | 25 | 12 (excessive motion) | 13 |
| **Tic** | 23 | 4  (2 MRI camera malfunction) (2 video recordings not permitting reliable tic identification) | 19 | 8 (excessive motion) | 11 |

*Note*. Two TS participants terminated the scanning session prior to the tic suppression condition. Control participants were matched one-to-one to TS participants using an automated procedure based on age, sex, handedness, IQ, and depressive symptomatology. Matched controls were excluded whenever the corresponding TS participant was excluded from a given analysis, resulting in comparable sample sizes and demographic distributions across the TS and typically developing control groups for each analysis.

**Table S2** Clinical and Demographic Characteristics of the TS Sample

| **Participant** | **Age** | **Sex** | **Tic Severity  (TTSS; 0-50)** | **Urge intensity  (PUTS; 9-36)** | **Subjective suppressibility (PUTS Item 10; 1-4)** | **Objective suppressibility (Percent reduction)** |
| --- | --- | --- | --- | --- | --- | --- |
| **1** | **7.07** | Male | 16 | 9 | 2 | 0.60 |
| **2** | **7.10** | Male | 7 | 17 | 1 | - |
| **3** | **8.01** | Male | 22 | 22 | 1 | 0.33 |
| **4** | **8.01** | Female | 34 | 25 | 1 | 0.90 |
| **5** | **8.04** | Male | 26 | 21 | 4 | - |
| **6** | **8.10** | Female | 28 | 15 | 4 | 0.71 |
| **7** | **9.01** | Male | 20 | 22 | 4 | - |
| **8** | **9.03** | Male | 19 | 20 | 3 | 0.08 |
| **9** | **9.04** | Male | 14 | 22 | 4 | 0.50 |
| **10** | **9.06** | Male | 21 | 9 | 3 | - |
| **11** | **9.07** | Male | 11 | 19 | 3 | - |
| **12** | **9.08** | Male | 22 | 11 | 1 | 0.61 |
| **13** | **10.01** | Male | 18 | 13 | 4 | 0.29 |
| **14** | **10.04** | Male | 9 | 21 | 3 | 0.71 |
| **15** | **10.12** | Male | 10 | 9 | 3 | - |
| **16** | **11.02** | Male | 16 | 24 | 3 | 0.75 |
| **17** | **12.01** | Male | 24 | 15 | 4 | 0.27 |
| **18** | **12.05** | Male | 23 | 27 | 3 | 0.67 |
| **19** | **12.09** | Male | 9 | 13 | 4 | 0.62 |
| **20** | **12.09** | Male | 12 | 20 | 2 | 0.91 |
| **21** | **12.09** | Female | 24 | 21 | 4 | 0.56 |
| **22** | **13.02** | Female | 19 | 22 | 4 | 0.50 |
| **23** | **15.06** | Female | 18 | 20 | 4 | 0.68 |
| **24** | **16.05** | Female | 12 | 26 | 3 | 0.70 |
| **25** | **16.07** | Male | 12 | 30 | 4 | 0.61 |

*Note*. Objective suppressibility parameters are missing for six participants. Two participants terminated the scanning session prior to the tic suppression condition. For two participants, tics could not be reliably evaluated based on video recordings. For two participants, technical issues with the camera setup resulted in lack of video recordings throughout scanning. TTSS = Total Tic Severity Score based on the Yale Global Tic Severity Scale [1, 2]. PUTS = Premonitory Urges for Tics Scale [3].

**Descriptive and Correlational Analyses of Clinical Measures**

The mean Total Tic Score (YGTSS) in the TS sample was 17.8 (SD = 6.7; range: 7–34), indicating overall mild to moderate tic severity. The mean PUTS score was 19.1 (SD = 6.0; range: 9–30), corresponding to medium urge intensity, as defined by the questionnaire manual (12.5–24.5). Based on established cut-offs, four participants (16%) reported low urge intensity (<12.5), including three who scored at the minimum value. Sixteen participants (64%) fell within the medium range, and five (20%) reported scores in the high range of urge intensity (25–30.5). No participants scored in the extreme range (>30.5). No significant correlation was observed between Total Tic Score (YGTSS) and premonitory urge intensity as measured by the PUTS (r = 0.039; P = 0.855). Age was not significantly correlated with either Total Tic Score (t_b_ = - 0.207; P = 0.153) or urge intensity (t_b_ = 0.159; P = 0.206).

**fMRI paradigm**

To examine neural correlates of voluntary urge suppression, we employed a suppression/release paradigm comprising three behavioural conditions: eye-blinking, yawning, and, for paediatric participants with TD, motor tics. Each condition consisted of six 36-second task blocks (three suppression, three release). During release blocks, participants were instructed to allow the urge-related behaviour to occur spontaneously. Conversely, during suppression blocks, they were instructed to inhibit the behaviour for the entire block duration. If an escape behaviour occurred (i.e., blink, yawn, or tic during suppression) [4, 5], participants were instructed to resume suppression immediately for the remainder of the block. Each block was preceded by a visual cue indicating the current task condition: a green traffic light for release and a red traffic light for suppression. After each suppression block, participants rated the perceived difficulty of suppression on a five-point visual analogue scale. Each rating period was followed by a 12-second baseline during which participants fixated on a central cross. The three conditions were always administered in a fixed order: blinking first, followed by yawning, and concluding with the tic condition.

To induce yawns, video clips depicting young actors or actresses yawning were presented during both release and suppression blocks. The contagiousness of these stimuli was validated in a pilot sample prior to the main study. Previous research has demonstrated that contagious yawning can be reliably elicited in children as young as 4–5 years [6-8]. To provide equivalent visual stimulation across blink-related blocks, video clips of individuals blinking were presented throughout all blink blocks. For each condition, six 12-second video clips were available. Within each block, three clips were shown in counterbalanced order, ensuring each appeared once in each serial position (first, middle, final). Stimuli included an equal number of male and female actors spanning the age range of the participant sample. Blink videos depicted only the eye region; yawn videos included the head and upper torso. In the tic condition, participants viewed personalized video recordings of themselves (6 × 36 seconds) taken immediately prior to scanning. These videos were acquired under standardized conditions (i.e., identical room, lighting, and setup) while participants were seated at a table and engaged in a preparatory video-viewing task. Each video depicted the participant’s head and upper body. Given that self-monitoring is known to increase tic frequency [9], these recordings were used to potentiate tic-related urges and optimize suppression opportunities throughout the task.

**Statistical Analysis of Difficulty Ratings**

***Within-domain analyses: Block-wise analyses of subjective difficulty.***

To assess potential temporal changes in perceived difficulty within each urge domain, repeated-measures ANOVAs were conducted on block-wise difficulty ratings. For blink and yawn, Block (1–3) was entered as within-subject factor and Group (TS, matched controls) as between-subject factor. For the tic domain, analyses were restricted to the TS group with Block (1–3) as within-subject factor. Sphericity was assessed using Mauchly’s test, and Greenhouse–Geisser correction was applied where appropriate. Linear and quadratic polynomial contrasts were additionally evaluated. For blink, no main effect of Block was observed, F(1.91, 82.16) = 0.56, p = .568, η²ₚ = .013, and the Block × Group interaction was not significant, F(1.91, 82.16) = 0.44, p = .635, η²ₚ = .010. Follow-up analyses within groups likewise revealed no significant Block effects in either the TS group, F(2, 42) = 0.46, p = .632, η²ₚ = .022, or controls, (F2, 44) = 0.54, p = .586, η²ₚ = .024. Polynomial contrasts were not significant in the TS group (linear: p = .381; quadratic: p = .906) or in matched controls (linear: p = 1.000; quadratic: p = .236). For yawn, the main effect of Block was not significant, F(1.84, 84.76) = 1.37, p = .259, η²ₚ = .029, but a significant Block × Group interaction emerged, F(1.84, 84.76) = 4.31, p = .019, η²ₚ = .086. Follow-up analyses within groups showed a significant effect of Block in the TS group, F(1.29, 28.29) = 4.30, p = .038, η²p = .164, with a significant linear component (F(1, 22) = 4.87, p = .038, η²ₚ = .181), whereas no Block effect was observed in matched controls, F(1.90, 45.64) = 0.94, p = .394, η²p = .038. For the tic domain (TS group only), there was no main effect of Block, F(1.85, 35.18) = 0.64, p = .523, η²ₚ = .033, and polynomial contrasts were not significant (linear: p = .481; quadratic: p = .367).

***Between-domain analyses: Difficulty across urge domains.***

To examine potential effects related to task sequence, repeated-measures ANOVAs were conducted on participant-level mean difficulty ratings averaged across blocks within each domain. First, analyses were restricted to the TS group and included all three domains (blink, yawn, tic) as a within-subject factor. A significant effect of Domain was observed, F(1.49, 26.78) = 11.36, p < .001, η²ₚ = .387. Polynomial contrasts revealed both linear (p = .013, η²ₚ = .297) and quadratic (p < .001, η²ₚ = .489) components. Second, to assess effects between the domains shared by both groups, a repeated-measures ANOVA was conducted, including Domain as a within-subject factor (blink, yawn) and Group as a between-subject factor. A significant main effect of Domain was found, F(1, 37) = 29.32, p < .001, η²ₚ = .442, whereas the Domain × Group interaction was not significant, F(1, 37) = 0.78, p = .382, η²ₚ = .021.

**Table S3** Descriptive block-wise subjective difficulty ratings across urge domains

| **Domain** | **Group** | **Difficulty Block1 (M ± SD)** | **Difficulty Block 2 (M ± SD)** | **Difficulty Block 3 (M ± SD)** |
| --- | --- | --- | --- | --- |
| **Blink** | **C** | 2.26 (± 1.4) | 2.09 (± 1.2) | 2.26 (± 1.3) |
| **Blink** | **TS** | 2.45 (± 1.3) | 2.55 (± 1.3) | 2.68 (± 1.2) |
| **Yawn** | **C** | 1.52 (± 1.0) | 1.36 (± 0.8) | 1.36 (± 0.6) |
| **Yawn** | **TS** | 1.13 (± 0.4) | 1.29 (± 0.6) | 1.57 (± 1.1) |
| **Tic** | **TS** | 1.60 (± 0.9) | 1.80 (± 1.0) | 1.75 (± 1.2) |

*Note*. Values represent group-level mean subjective difficulty ratings (± SD) for each block and domain. The table provides a descriptive illustration of within-domain patterns and differences across urge domains. TS = participants with chronic tic disorders; C = typically developing controls. M = Mean; SD = Standard deviation.

**Neural substrates of blink suppression**

When escape events were retained, blink suppression in TS patients (n = 16) was associated with a significant right-hemispheric cluster (x, y, z = 60, 8, 10; k_E =_ 1036; pseudo t = 5.13; P_FWE-corr._ = 0.019) and corresponding left-hemispheric cluster (x, y, z = -34, 16, 12; k_E =_ 953; pseudo t = 4.63; P_FWE-corr._ = 0.021) including the inferior frontal gyrus pars opercularis, frontal operculum cortex (OP8), dorsal anterior insula (Id6), and precentral gyrus. Additional activations emerged in the inferior parietal lobule (areas PF, PFm, PFt, PFop): (x, y, z = 66, -18, 12; k_E =_ 427; pseudo t = 4.35; P_FWE-corr._ = 0.048) and in the right middle frontal gyrus, extending into the frontal pole (x, y, z = 38, 42, 30; k_E =_ 553; pseudo t = 5.04; P_FWE-corr._ = 0.037; see Figure S1). Removing escape events from suppression blocks yielded significant right hemispheric activations only: in a cluster including the inferior frontal gyrus pars opercularis, frontal operculum cortex (OP8), dorsal anterior insula (Id6), and precentral gyrus (x, y, z = 36, 16, 10; k_E =_ 808; pseudo t = 4.74; P_FWE-corr._ = 0.026) and in a cluster centred in the middle frontal gyrus, extending into the frontal pole (x, y, z = 38, 44, 30; k_E =_ 486; pseudo t = 4.86; P_FWE-corr._ = 0.047).

When escape events were retained, blink suppression in typically developing children and adolescents (n = 51) was associated with significant activation in a right-hemispheric cluster (x, y, z = 44, 14, -6; k_E =_ 1287; pseudo t = 5.61; P_FWE-corr._ = 0.007) and corresponding left-hemispheric cluster (x, y, z = -34, 12, 12; k_E =_ 1843; pseudo t = 6.17; P_FWE-corr._ = 0.004) including the inferior frontal gyrus pars opercularis, frontal operculum cortex (OP8), and dorsal anterior insula (Id6). Additional activations emerged in the frontal pole/middle frontal gyrus bilaterally (left: x, y, z = -36, 52, 24; k_E =_ 252; pseudo t = 3.85; P_FWE-corr._ = 0.045; right: x, y, z = 38, 48, 30; k_E =_ 389; pseudo t = 5.04; P_FWE-corr._ = 0.030) and in midline regions including SMA and pre-SMA (x, y, z = 2, 2, 60; k_E =_ 236; pseudo t = 4.91; P_FWE-corr._ = 0.048; see Figure S1). Removing escape events from suppression blocks yielded significant activations in only two clusters involving the inferior frontal gyrus pars opercularis, frontal operculum cortex (OP8), and dorsal anterior insula (Id6) bilaterally (left: x, y, z = -34, 14, 12; k_E =_ 350; pseudo t = 5.28; P_FWE-corr._ = 0.036; right: x, y, z = 44, 14, -6; k_E =_ 539; pseudo t = 5.01; P_FWE-corr._ = 0.023).

**Neural substrates of yawn suppression**

In patients with TS (n = 13), yawn suppression was associated with widespread activation encompassing frontal, parietal, temporal, and midline regions. The largest cluster (x, y, z = –52, 26, 0; kE = 9392; pseudo-t = 6.54; _FWE-corr._ < 0.001) included the frontal operculum (OP8), inferior frontal gyrus (pars triangularis/Area 45), middle frontal gyrus, and dorsal anterior cingulate cortex (Area p32). Bilateral parietal activations emerged in the superior parietal lobule (Area 7A), intraparietal sulcus (hIP3, hIP6), inferior parietal lobule (Areas PGa, PFm), and lateral occipital cortex on the left (x, y, z = –36, –62, 60; kE = 2049; pseudo-t = 5.28; P_FWE-corr._ = 0.002) and in homologous right-hemispheric regions including angular gyrus (x, y, z = 34, –56, 46; kE = 1144; pseudo-t = 5.12; P_FWE-corr._ = 0.003). Additional activations were found in the precuneus (Area 7P; x, y, z = 0, –70, 46; kE = 432; pseudo-t = 4.67; P_FWE-corr._ = 0.007), in left posterior middle/superior temporal cortex (Areas TE2–5; x, y, z = –66, –34, 0; kE = 1124; pseudo-t = 6.00; P_FWE-corr._ = 0.003), in right posterior middle/superior temporal cortex (Area TE5; x, y, z = 66, –28, –6; kE = 378; pseudo-t = 4.92; P_FWE-corr._ = 0.008), and in a smaller right superior temporal cluster encompassing planum temporale and TE3 (x, y, z = 66, –22, 12; kE = 139; pseudo-t = 4.62; P_FWE-corr._ = 0.018; see Figure S1).

In typically developing children and adolescents (n = 39) yawn suppression was associated with widespread activation encompassing frontal, parietal, temporal, occipital, midline, and cerebellar regions. The largest cluster (x, y, z = 2, –80, -30; kE = 2713; pseudo-t = 4.95; P_FWE-corr._ = 0.003) was centred in the cerebellum. Robust posterior activation also emerged in lateral occipital cortex/occipital fusiform gyrus extending into posterior supramarginal and superior temporal gyrus (x, y, z = -40, –82, -14; kE = 2357; pseudo-t = 5.14; P_FWE-corr._ = 0.004). Further engagement was observed in the superior parietal lobule together with lateral occipital cortex (x, y, z = 20, –64, 64; kE = 1120; pseudo-t = 4.29; P_FWE-corr._ = 0.007) and in motor-executive regions including dorsal premotor cortex (areas 6d1/6d2) and precentral gyrus (x, y, z = 24, –8, 74; kE = 2260; pseudo-t = 4.91; P_FWE-corr._ = 0.004). Additional frontal engagement comprised a left frontal pole/inferior frontal gyrus cluster (areas 44/45) cluster (x, y, z = -48, 40, -6; kE = 600; pseudo-t = 4.64; P_FWE-corr._ = 0.013) and a midline frontal pole/superior frontal gyrus cluster (x, y, z = 0, 38, 58; kE = 773; pseudo-t = 4.20; P_FWE-corr._ = 0.010; see Figure S1).

**Fig. S1 Task recruitment (natural urge suppression)**

*Note*. **(a)** Patients with TS. Blink suppression (left; escape events retained; n=16) and yawn suppression (right; escape events removed; n=13). **(b)** Typically developing cohort**.** Blink suppression (left; escape events retained; n=51) and yawn suppression (right; escape events removed; n=39). Maps show activation for suppression > release. Rendered on a canonical brain (MRIcroGL) [10]. Colour bars indicate pseudo-t values (test statistic from nonparametric permutation testing implemented in SnPM13); L = left, R = right. These panels document task recruitment within each group (not between-group contrasts).

**Fig. S2 Exploratory whole-brain PPI effects**

*Note*. **Top:** Seed = right IPL/angular gyrus from the yawn patient > control contrast. Stronger coupling between this seed and left extrastriate-visual cortex and cerebellar crus were negatively associated with tic-suppression success. Activations are rendered on canonical brains (MRIcroGL) [10]. **Bottom:** Scatterplots**:** each data point represents one participant. Solid lines = least-squares fit; dashed lines = 95% confidence intervals. Colour bar indicates pseudo-t values (test statistic from nonparametric permutation testing implemented in SnPM13). L = left; R = right.

**References**

1.	Leckman JF, Riddle MA, Hardin MT et al. (1989) The Yale Global Tic Severity Scale: Initial testing of clinician-rated scale of tic severity. *J Am Acad Child and Adolesc Psychiatry* 28:556-573. <https://doi.org/10.1097/00004583-198907000-00015>

2.	Storch EA, Murphy TK, Geffken GR, Sajid M, Allen P, Roberti JW (2005) Reliability and validity of the Yale global tic severity scale. *Psychol. Assess* 17:486-491. <https://doi.org/10.1037/1040-3590.17.4.486>

3.	Openneer TJC, Tárnok Z, Bognar E et al. (2020) The Premonitory Urge for Tics Scale in a large sample of children and adolescents: psychometric properties in a developmental context. An EMTICS study. Eur Child Adolesc Psychiatry 29:1411-1424. <https://doi.org/10.1007/s00787-019-01450-1>

4.	Abi-Jaoude E, Segura B, Cho SS, Crawley A, Sandor P (2018) The Neural Correlates of Self-Regulatory Fatigability During Inhibitory Control of Eye Blinking. J Neuropsychiatry Clin Neurosci 30:325-333. <https://doi.org/10.1176/appi.neuropsych.17070140>

5.	Bhikram T, Crawley A, Arnold P, Abi-Jaoude E, Sandor P (2021) Neuroimaging the emotional modulation of urge inhibition in Tourette Syndrome. Cortex 135:341-351. <https://doi.org/10.1016/j.cortex.2020.10.010>

6.	Anderson JR, Meno P (2003) Psychological influences on yawning in children. Current psychology letters. Behaviour, brain & cognition. <https://doi.org/10.4000/cpl.390>

7.	Helt MS, Eigsti IM, Snyder PJ, Fein DA (2010) Contagious yawning in autistic and typical development. Child Dev 81:1620-1631. <https://doi.org/10.1111/j.1467-8624.2010.01495.x>

8.	Hoogenhout M, Van Der Straaten K, Pileggi L, Malcolm-Smith S (2013) Young children display contagious yawning when looking at the eyes. J. Child Adolesc. Behav. <https://doi.org/10.4172/jcalb.1000101>

9.	Brandt VC, Lynn MT, Obst M, Brass M, Münchau A (2015) Visual feedback of own tics increases tic frequency in patients with Tourette's syndrome. Cogn Neurosci 6:1-7. <https://doi.org/10.1080/17588928.2014.954990>

10.	Rorden C (2025) MRIcroGL: voxel-based visualization for neuroimaging. Nature Methods 22:1613-1614. <https://doi.org/10.1038/s41592-025-02763-7>
